# Topotecan Delivery to the Optic Nerve after Ophthalmic Artery Chemosurgery

**DOI:** 10.1371/journal.pone.0151343

**Published:** 2016-03-09

**Authors:** Paula Taich, Flavio Requejo, Marcelo Asprea, Mariana Sgroi, Pierre Gobin, David H. Abramson, Guillermo Chantada, Paula Schaiquevich

**Affiliations:** 1 Clinical Pharmacokinetics Unit, Hospital de Pediatría JP Garrahan, Buenos Aires, Argentina; 2 National Scientific and Technical Research Council, CONICET, Buenos Aires, Argentina; 3 Service of Interventional Radiology, Hospital de Pediatría JP Garrahan, Buenos Aires, Argentina; 4 Animal facility, Laboratory, Hospital de Pediatria J.P. Garrahan, Buenos Aires, Argentina; 5 Service of Ophthalmology, Hospital de Pediatría JP Garrahan, Buenos Aires, Argentina; 6 Ophthalmic Oncology Service, Memorial Sloan-Kettering Center Cancer Center, New York, United States of America; 7 Research Institute at Hospital de Pediatría JP Garrahan, Buenos Aires, Argentina; University of Kentucky, UNITED STATES

## Abstract

Extraocular retinoblastoma is a major challenge worldwide, especially in developing countries. Current treatment involves the administration of systemic chemotherapy combined with radiation, but there is a clear need for improvement of chemotherapy bioavailability in the optic nerve. Our aim was to study the ophthalmic artery chemosurgery (OAC) local route for drug delivery assessing ocular and optic nerve exposure to chemotherapy and to compare it to exposure after intravenous infusion (IV) of the same dose in an animal model. Topotecan was used as a prototype drug that is active in retinoblastoma and based on the extensive knowledge of its pharmacokinetics in preclinical and clinical settings. Five Landrace pigs received 4mg of topotecan via OAC as performed in retinoblastoma patients. At the end of the infusion, the eyes were enucleated, the optic nerve and retina were dissected, and the vitreous and plasma were separated. After recovery and a wash-out period, the animals received a 30-min IV infusion of topotecan (4 mg). The remaining eye was enucleated and tissues and fluids were separated. All samples were stored until quantitation using HPLC. A significantly higher concentration of topotecan in the optic nerve, vitreous, and retina was obtained in eyes after OAC compared to IV infusion (p<0.05). The median (range) ratio between topotecan concentration attained after OAC to IV infusion in the optic nerve, retina and vitreous was 84(54–668), 143(49–200) and 246(56–687), respectively. However, topotecan systemic exposure after OAC and IV infusion remained comparable (p>0.05). The median optic nerve-to-plasma ratio after OAC and IV was 44 and 0.35, respectively. Topotecan OAC delivery attained an 80-fold higher concentration in the optic nerve compared to the systemic infusion of the same dose with similar plasma concentrations in a swine model. Patients with retinoblastoma extension into the optic nerve may benefit from OAC for tumor burden by increased chemotherapy bioavailability in the optic nerve without increasing systemic exposure or toxicity.

## Introduction

Extraocular retinoblastoma is still a clinical problem in developing countries [1,2]. In children with overt extraocular disease and especially those with central nervous system (CNS) invasion, the overall survival rate is significantly lower than in patients with intraocular disease [1]. The optic nerve is a major route of dissemination of retinoblastoma [3]. Optic nerve invasion is usually present in eyes with advanced disease which can be classified as International Classification of Retinoblastoma (IBRb) Group E [4]. Early optic nerve invasion is manifested only microscopically and it is important for predicting extraocular relapse when the tumor extends beyond the lamina cribrosa in enucleated eyes [5]. If untreated, massive invasion of the optic nerve usually occurs, causing obvious enlargement of the optic nerve on imaging studies and leading to direct spread to the CNS [6]. In these cases, the tumor may also disseminate by leptomeningeal seeding. Molecular studies have shown that minimally disseminated disease in the cerebrosinal fluid (CSF) is significantly more common in eyes with massive optic nerve invasion and glaucoma and molecular alterations can be found before clinical signs of disease spread [7]. Hence, in many cases, the tumor invades the optic nerve and from there it seeds the CSF molecularly before clinical detection. Therefore, early aggressive treatment would be needed to improve survival [8–10,12].

On some occassions, the optic nerve invasion is so extense that a tumor remnant in the optic nerve stump may be left behind after enucleation [11]. These children need intensive therapy because of this microscopical residue with direct access to the CNS [12]. Standard imaging studies traditionally had limited sensitivity and specificity for detecting microscopical optic nerve invasion [13,14] and only when the optic nerve is massively enlarged many centers use neoadjuvant systemic chemotherapy in order to provide anticancer treatment immediately, postponing enucleation for two or three months [6]. Even with this intensive strategy, most patients with massive optic nerve involvement do poorly and CSF relapse usually occurs after an initial period of tumor response [4]. Hence innovative alternatives are needed for the treatment of these children.

The advent of ophthalmic artery chemosurgery (OAC) changed the treatment paradigm for intraocular disease but, to our knowledge, its use in extraocular disease has not been reported [15].

Infusion of topotecan by means of OAC has proved to increase bioavailability in the eye compared to the periocular injection in the swine model while showing a favorable ocular-to-plasma exposure ratio [16]. Thus, a reduced number and severity of systemic adverse events should be expected. Nonetheless, the exposure to chemotherapy in the optic nerve after OAC or intravenous infusion (IV) has never been characterized, although the latter is the current route for drug delivery for extraocular disease [8,10,12]. Taking into account that the ophthalmic artery supplies the blood to the optic nerve in its orbital segment, we hypothesised that increased levels of chemotherapy may also be achieved in the optic nerve after OAC compared to the IV infusion. Besides, patients with optic nerve involvement would also benefit from higher ocular disposition of drugs during preoperative chemotherapy to facilitate the enucleation and make it a safer procedure [17].

Topotecan is an active agent against retinoblastoma [18]. Our group has vast experience with this agent in the clinics as well as an extensive analytical background in the quantification of topotecan in several ocular structures. In addition, we previously characterized topotecan pharmacokinetics after several routes of drug delivery [19]. Despite topotecan is not a first-line agent in retinoblastoma treatment like melphalan, the latter is not systemically administered in retinoblastoma due to its severe myelotoxicity [20]. Thus, we selected topotecan as a candidate for the present study and considered it as a proof of concept for potential translation of the results to other chemotherapeutic agents. Moreover, in line with all other chemotherapy treatments in oncology, we studied topotecan OAC infusion of a high dose as a potential route for drug delivery specifically to the optic nerve as part of other concomitant current treatments.

Therefore, the aim of the present study was to compare topotecan concentration in the optic nerve, plasma, and intraocular structures of a swine model after OAC to levels attained after IV infusion for potential translation of this route for administration to patients with retinoblastoma and invasion to the optic nerve as a proof of concept that may also be applied to other chemotherapy agents.

## Materials and Methods

### Ethics statement

The present study was approved by Coordinación de Investigación Hospital de Pediatría JP Garrahan (protocol number 861) and conducted complying with the tenets of the Association for Research in Vision and Ophthalmology for the use of animals in ophthalmic and vision research. The institutional animal care committee approved the use of both eyes of each animal detailed in the submitted protocol.

### Chemicals and reagents

Topotecan hydrochloride analytical standard was donated by Asofarma S.A. (Buenos Aires, Argentina). Stock solutions of topotecan were prepared in methanol and stored at -20C to minimize degradation. Topotecan used for pig studies was obtained from Microsules (Buenos Aires, Argentina). HPLC-grade water was obtained using a Milli-Q system (Millipore Corporation (Billerica, MA). Acetonitrile, triethilamine and methanol were high-pressure liquid chromatography (HPLC) grade and glacial acetic acid was of analytical grade also obtained from Sintorgan (Buenos Aires, Argentina).

### Topotecan administration and sampling schedule

In the present study we used five pigs (Landrace, *Sus scrofa domestica*) weighing 25–30 kg. The animals were sedated and maintained under mechanical ventilation [16,21].

Sedation was attained using an intramuscular injection of 20 mg/kg ketamine (Inducmina^®^, Dr Gray, Argentina) in combination with 0.8 mg/kg midazolam (Midazolam Gemepe^®^, GEMEPE, Argentina) and 0.1 mg/kg atropine (Norgreen S.A, Argentina). Afterwards, general anesthesia was induced with an intravenous injection of 2.2 mg/kg of propofol (Dubernard^®^, Northia, Argentina) and maintained under mechanical ventilation with isoflurane 2% (Baxter Healthcare, Puerto Rico) and 2μg/kg fentanyl (Denver Farma, Argentina) and 0.1 mg/kg pancuronium (Bemicin^®^, Northia, Argentina). Concomitant to general anesthesia, the animals received normal saline through the marginal ear vein. Body temperature and cardiorespiratory parameters were monitored continuously using a rectal temperature probe, an external ECG, and a pulse oximeter, throughout anesthesia. Body temperature was maintained using an external heat source forced air unit (Warm Touch, Mallinckrodt, Medical).

Each animal was used on two occasions and all animals were allocated to the same sequence of drug treatment. On the first day, OAC was performed as previously described and according to the vascular anatomy of the swine model [16,22,23]. Briefly, under heparin (75 UI/kg) anticoagulation the femoral artery was accessed by percutaneous puncture using a 16G needle in the point where an imaginary line traced from the last nipple crosses perpendicularly the inguinal ligament. By means of the Seldinger technique, a 5-French vascular sheath (Johnson & Johnson, Cordis^®^, USA) was placed in a femoral artery of the animal. Then, a 5-French guide catheter (Stryker Neurovascular, Fremont, CA) was advanced through the aortic arch and positioned in the left or right common carotid artery and thereafter the ophthalmic artery was super-selectively catheterized using a microcatheter (Marathon^®^, Irvine, CA, USA) over a guidewire (Mirage^®^, Irvine, CA, USA). Afterwards, 4mg of topotecan (Topotecan Microsules^®^, Microsules, Argentina) in 30 ml of saline were infused into the ophthalmic artery in a pulsatile fashion over 30 minutes as currently used in the clinics of retinoblastoma patients [22].

A dose of 4 mg was selected as it is a higher amount than that used in clinical practice for OAC but lower than the total systemic dose calculated according to the body surface area if topotecan is daily infused over a 30-minute interval for 5 consecutive days for each of two consecutive weeks based on current clinical protocols (2 mg/m^2^/d) [8,15,24]. The currently used dose of topotecan after OAC is between 1 and 2 mg. However, based on the size of the animal model and therefore the volume of distribution, this dose would have been too low to attain any detectable concentration in the ocular tissues after the intravenous dose thus supporting the use of 4 mg.

Blood samples from the femoral artery were collected in a pre-heparinized tube 15 minutes after the end of the infusion. Then, the treated eye was enucleated 30 minutes after the end of the infusion using curved scissors and an enucleation spoon in order to preserve the longest portion of the globe section of the optic nerve. Finally, tarsorrhaphy was performed by a trained ophthalmologist using Nylon 2.0 suture (Mononylon 2.0, Ethicon) in order to avoid a potential focus of infection. Based on the experience of our previous studies that we collected samples using the microdialysis technique we observed that the maximum vitreous concentration was attained during the first 30-minute interval after the end of the OAC infusion] [16]. Moreover, the enucleation technique requires at least 15 minutes. Altogether, we collected the vitreous sample 30 minutes after the end of the infusion as a surrogate of the maximum topotecan concentration. Thereafter, the retina was dissected and both the retina and optic nerve were washed with cold saline solution while the vitreous was also isolated. All samples were stored in a 1.5 ml tube on ice until processing. The animal was recovered from the anesthesia and treated with cephalexin at a dosage of 60 mg/kg IM every 12 hours for five days (Ruminal, Argentina). In order to prevent animal pain associated with the surgical procedure, subcutaneous administration of carprofen 4mg/kg/day (Caroprobay^®^, Bayer Animal Health, Argentina) was provided for 72 hours concomitant with buprenorphine 0.04 mg/kg SC every 8 hours for 24 hours after the procedure (Lafedar, Argentina). During the immediate postoperative period, animals were cleaned and frequently monitored (respiratory and cardiovascular function, body temperature) by trained veterinarians. Animals were monitored every 8 hours for clinical signs of pain or distress and thereafter they were checked every 12 hours for general state, feeding behavior, nasal or ocular pathological secretions and the enucleation wound was supervised. Lastly, hematological controls were performed the day before surgery and 72 hours and 7 days after surgery. After a washout period of 10 days, the same animal was subjected to a second pharmacokinetic study in the fellow eye after the end of a 30-minute intravenous infusion of the same dose of topotecan. Topotecan was infused through the marginal ear vein of the animals. Plasma sample collection and enucleation for tissue dissection and vitreous isolation were carried out as previously described for the pharmacokinetic study after OAC [16,25]. Finally, after the second pharmacokinetic study the anesthetized animal was euthanized with a dose of 1 meq/Kg of potassium chloride (Norgreen, Argentina). Altogether, from the start of animal sedation until euthanasia the second study lasted about 3 hours. Thus, both eyes of the same animal were used in the present study and each of them served as comparison to the other and was studied only once per occasion.

### Sample processing and topotecan analytical assay

Blood samples were centrifuged and plasma was separated and precipitated with cold methanol/HCL (10:1) in order to quantitate total topotecan [16]. At each pharmacokinetic study, after the eye was enucleated the optic nerve was cut in two pieces. Specifically, the proximal segment of 1 cm-length measured from the optic nerve head was separated from the distal portion of the isolated nerve. Additionally, the retina was dissected and both the optic nerve and the retina were washed with cold PBS, weighed, and homogenized with cold acidic methanol in a 1-to-5 dilution using IKA T25 Ultra-Turrax in a cold bath. During tissue homogenization, transient and local rise in temperature can accelerate topotecan interconversion between the active lactone to the carboxylic form. Therefore, we decided to measure total topotecan instead of the quantifying a potentially inaccurate concentration of the lactone form. All methanolic supernatant extracts were stored at -20°C until assay.

Topotecan concentrations were determined by HPLC coupled with fluorometric detection. Briefly, the analysis was performed with an Agilent HPLC system (Buenos Aires, Argentina) equipped with an Agilent 1100 liquid chromatography pump and an Agilent 1260 fluorescence detector set at an excitation/emission wavelength of 370nm and 530nm, respectively. Separation chromatography was performed using a Nova-pack C18 reverse-phase column (150 mm x 3.9 mm i.d., 4 μm particle size; Waters, Milford, United states) coupled to a C18 Penomenex security guard pre-column. Data acquisition and processing was performed using the Agilent ChemStation software. The lower limit of quantitation was 1 ng/mL and the intra and inter-day precision was 7%. The linear ranges for plasma, vitreous humor, and optic nerve assays were from 1 to 600ng/ml, respectively. Samples were diluted if needed and dilutions were validated. Data under the limit of quantitation were assumed as 1 ng/ml.

### Statistical Analysis

Topotecan concentrations in the different tissues were compared between groups by means of the non-parametric Wilcoxon signed rank test and examined for significance at a level of p≤0.05 using R [26].

## Results

### Animal samples

A total of five animals were included in the present study. All animals were studied on two occasions, with a first pharmacokinetic study after OAC and a second after IV infusion of the same dose of topotecan. Thus, 10 pharmacokinetic studies were available for analysis and a total of 49 samples of proximal and distal optic nerve, vitreous, plasma, and retina were obtained for pharmacokinetic analysis. One proximal optic nerve sample obtained after OAC was lost during sample processing.

Total topotecan concentrations in the proximal and distal optic nerves, retina, vitreous, and plasma after OAC and IV infusion are shown in Table 1.

**Table 1:** Topotecan concentration in plasma and ocular tissues after ophthalmic artery chemosurgery and intravenous infusion of 4 mg in the pig.

Tissue or biological fluid	Topotecan concentration after OAC	Topotecan concentration after IV	Ratio topotecan concentration OAC/IV
**Proximal optic nerve (ng/g tissue)**	2633 (1528–3847)*	13.6 (1.0–70.7)	84 (54–668)
**Distal optic nerve (ng/g tissue)**	1864 (1768–6718)* ‡	20.8 (1.0–74.4) ‡	275 (25–1768)
**Retina (ng/g tissue)**	4286 (3552–11434) *‡	64.7 (30.0–84.7) ‡	143 (49–200)
**Vitreous (ng/ml)**	1237 (246–1371) *	1.8 (1.0–22.1)	246 (56–687)
**Plasma (ng/ml)**	72.7 (51.6–98.0) †	49.2 (31.9–74.2)	1.8 (0.8–2.2)

Data are shown as median (range)

*p≤0.05 compared with the pharmacokinetic parameters obtained in the same tissue after intravenous infusion.

^†^ p>0.05 compared with the pharmacokinetic parameters obtained in the same tissue after intravenous infusion.

^‡^p>0.05 compared with topotecan concentration in the proximal optic nerve.

**Abbreviations**: OAC, ophthalmic artery chemosurgery; IV, intravenous infusion.

### Topotecan pharmacokinetics after ophthalmic artery chemosurgery

As shown in Table 1, ophthalmic artery chemosurgery led to comparable topotecan concentrations in the proximal and distal optic nerve (p>0.05). Moreover, there was no significant difference in the concentration of the chemotherapeutic agent in the optic nerve compared to that attained in the retina although a trend towards higher levels in the retina was found (p>0.05). Finally, median (range) topotecan plasma concentration attained 0.25 hours after the end of the OAC infusion was 72.7ng/ml (51.6–98.0) as shown in Table 1.

### Topotecan disposition after endovenous infusion

Similar to the results obtained after OAC, topotecan IV infusion led to a homogeneous concentration along the optic nerve without significant difference between the exposure in the proximal optic nerve and that in the distal portion (p>0.05). However, in both distal and proximal optic nerve segments topotecan levels were similar at only 15 ng/g tissue (Table 1). Moreover, retina and plasma topotecan concentrations attained after IV infusion were all in the same order of magnitude and around 50ng/ml while the median (range) vitreous concentration was as low as 2ng/ml (1–22) (Table 1). In line with the observations after OAC, retina and proximal optic nerve concentrations after IV infusion were not statistically different, despite a trend towards lower concentrations was found in the optic nerve (p>0.05).

### Comparison of topotecan ocular concentrations after OAC and IV routes

**Fig 1:**
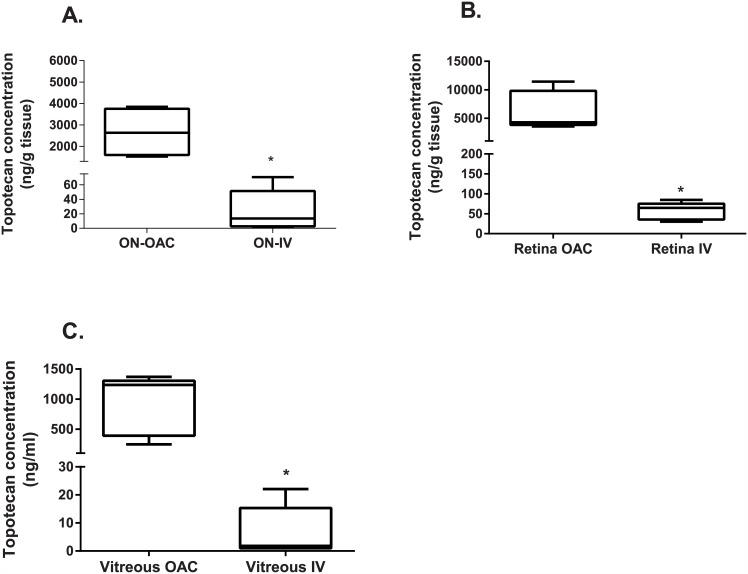
Topotecan concentration in the (A) optic nerve, (B) retina, and (C) vitreous after ophthalmic artery chemosurgery compared to intravenous infusion. * Topotecan levels were statistically different between groups (p<0.05).

We observed a significant greater topotecan exposure in the optic nerve after OAC compared to the concentrations attained after IV infusion (Fig 1A) (Table 1, p≤0.05). Specifically, the median (range) proximal and distal optic nerve ratio between OAC and IV topotecan delivery was 84 (54–668) and 275 (25–1768), respectively. Thus, OAC delivery of topotecan allowed attaining at least 80 times the concentration found after IV in the optic nerve. Moreover, the retina and vitreous exposure ratio between the two studied routes of drug delivery also showed a significant difference as shown in Fig 1B and 1C (p<0.05). Therefore, topotecan exposure in the retina and vitreous of those eyes in which the agent was delivered through OAC was 143 and 246 times greater than after IV infusion. Conversely, there was no statistical difference in topotecan blood levels after both routes of drug delivery (Table 1, p>0.05).

Altogether, OAC lead to higher concentrations in the optic nerve (ON), vitreous, and retina while attaining similar plasma exposure after OAC compared to IV infusion of the same dose of 4 mg of topotecan.

### Local versus systemic topotecan exposure

**Fig 2:**
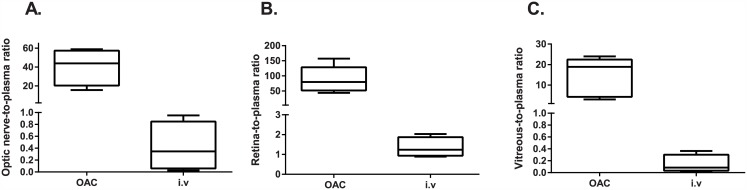
Ocular-to-plasma topotecan concentrations attained after ophthalmic artery chemosurgery and intravenous infusion. (A) Optic nerve-to-plasma concentrations after OAC and IV, (B) retina-to-plasma levels after OAC and IV, (C) and vitreous-to-plasma topotecan concentrations after both routes of drug delivery.

Of note was that topotecan delivery through OAC was not only the best route for delivering high levels to the ocular tissues and optic nerve of the animal, but also a more selective route to attain the ocular tissues with respect to the systemic exposure. In this sense, the median ratio between the proximal optic nerve-to-plasma, retina-to-plasma, and vitreous-to-plasma topotecan levels after OAC was between 13 and 70 implying that these tissues are exposed to more than 10 times the systemic exposure (Fig 2A, 2B and 2C; Table 2). Conversely, topotecan exposure in the optic nerve, vitreous, and retina was about the same or much lower than plasma concentrations after IV infusion demonstrating the lack of selectivity to expose the ocular tissues to chemotherapy of this route of drug delivery (Table 2).

**Table 2:** Ocular tissues to plasma ratios according to the route of drug delivery.

Tissues or fluids ratio, route of delivery	Median ratio (range)
Optic nerve-to-plasma, OAC	44 (16–59)
Retina-to-plasma, OAC	70 (44–100)
Vitreous-to-plasma, OAC	13 (3–24)
Optic nerve-to-plasma, IV	0.35 (0.03–0.95)
Retina-to-plasma, IV	1.24 (0.89–2.03)
Vitreous-to-plasma, IV	0.09 (0.03–0.36

**Abbreviations**: OAC, ophthalmic artery chemosurgery; IV, intravenous infusion.

## Discussion

The present study is the first to assess the concentrations of topotecan in the optic nerve attained after OAC and suggests this as a potential alternative route of chemotherapy delivery for retinoblastoma patients with optic nerve invasion. In addition, we characterized the ocular and systemic levels after IV infusion of topotecan in a swine model and compared the concentrations with those attained after OAC with potential implications for patients with optic nerve involvement that undergo preoperative chemotherapy for tumor reduction.

Even though OAC has gained a prominent role in the conservative treatment of retinoblastoma, its use for patients with retinoblastoma and optic nerve extension has not been proposed before. Massive optic nerve invasion is considered as Stage III disease according to the International Retinoblastoma Staging System and these patients are usually treated with intensive systemic neoadjuvant therapy followed by secondary enucleation or limited exenteration, adjuvant systemic chemotherapy, and orbital radiotherapy [6,8,9,27]. Even with this intensive approach, the survival of these children is around 20–30%. Most children succumb to leptomeningeal dissemination after a short initial period of clinical response. Recent studies from our group have shown that minimal seeding of the CSF occurs more frequently in children with massive optic nerve invasion and glaucoma in the absence of systemic dissemination, suggestive of direct tumor seeding to the CSF from the optic nerve [7]. In addition, our studies show that when CSF relapse occurs, no evidence of disease is found in the bone marrow at a molecular level [28]. Based on these data, we hypothesize that it may be clinically advantageous to improve the delivery of chemotherapy drugs to the optic nerve in an attempt to cause an immediate and massive response locally in the optic nerve and potentially reduce the occurrence of seeding to the CSF. Moreover, higher bioavailability in the ocular structures of metastatic patients may enhance tumor reduction and thereby, facilitate the enucleation procedure and resection of an adequate optic nerve stump [12,17].

Since there are no previous reports on chemotherapy distribution along the optic nerve, we characterized the concentration of topotecan in two segments of the tissue in order to determine whether the drug can achieve concentrations differently along the orbital segment of the nerve. We found that the topotecan level in the proximal and distal optic nerve was similar in the swine model both after OAC and IV infusion. These findings could have implications for the clinical management of retinoblastoma patients with disseminated disease. Whether the tumor is proximal or more distal to the resection margin, it may all be targeted in the orbital section by chemotherapy delivered using OAC. In the present report it is conclusively shown that topotecan delivery by IV infusion, although attaining similar concentrations in the proximal and distal segments, it reached levels that were significantly lower than those obtained using OAC (p<0.05). After OAC, topotecan concentration was at least 80 times higher than after systemic infusion. Therefore, based on the present findings we reinforce the concept that exposure of the optic nerve to chemotherapy is increased using OAC compared to the IV infusion. Whether the large difference observed in topotecan exposure in the optic nerve after OAC compared to IV infusion remains the same after other than the currently used dose should be evaluated in further studies. Moreover, topotecan median concentration in the optic nerve was about 2,000 ng/g tissue. If considering a density of the tissue equal to water (1g/ml), the attained concentration would be about 2,000ng/ml, about 140-fold times the concentration required to inhibit 50% of the cell culture (IC50, 14ng/ml or 30nM based on previous reports)[18]. On the contrary, topotecan concentration in the optic after IV infusion was only about 20ng/g tissue, almost the IC50 if the previously explained assumptions are taken into account. In the present study our aim was to characterize the concentration of topotecan in the optic nerve of the swine model after OAC and to compare the exposure attained in this tissue after IV infusion as the latter is the route of choice for retinoblastoma patients with invasion to the optic nerve. Thus, our results may be used as a basis for further clinical studies and the pharmacologically active dose (above the IC50) should be assessed in the corresponding efficacy studies.

Several previous reports on the experience of topotecan dose adjustment in children with cancer have shown a wide inter-patient variability in topotecan disposition with an up to 12-fold range in the clearance of this agent after intravenous infusion of a fixed dose. Researchers at St Jude Children’s Research Hospital have published extensively on the feasibility and utility of using a pharmacokinetically guided approach to control this variability and individualize topotecan dosage according to the pharmacokinetic behavior of each specific patient. Therefore, a high inter-individual variability in systemic and ocular tissues concentrations was expected in our study as shown in Table 1. On the contrary, much less variability was observed in optic nerve and ocular tissues topotecan concentrations favoring to the use of OAC for targeting these structures [29,30].

Current treatment of patients with disseminated retinoblastoma and tumor at the resection margin consists of IV infusion of high doses of chemotherapy. The selection of a route of administration is not only based on the amounts of drug that can be delivered to the target tissue, but also on the selectivity with respect to the exposure in normal tissues. In the present study we showed that the median optic nerve-to-plasma exposure after OAC was 44 times whereas after IV infusion it was less than 0.5. Moreover, both routes of drug administration led to comparable topotecan systemic exposure. Therefore, systemic chemotherapy is not only ineffective for achieving high concentrations in the optic nerve, but it also lacks selectivity to target the tissue with tumor while exposing the rest of the body to unnecessary levels of chemotherapy.

Although there was a trend towards higher topotecan levels in the retina than those attained in the optic nerve, the difference was not statistically significant both after OAC and IV infusion. This finding could be explained by the common artery that irrigates both structures. In the pig, the retina is supplied with blood from the ciliary arteries that branch from the ophthalmic artery while the optic nerve is irrigated by pial vessels and branches of the ophthalmic artery that derive in six lateral retinal arteries that run in the periphery of the nerve [31]. Thus, it was expected that topotecan levels would be similar between tissues although the local mechanism of drug elimination may vary. In addition, this is the first report to show that topotecan levels in the retina were about 140 times higher after OAC than after IV infusion. This result may explain the efficacy observed in the clinics in eyes with extensive retinal tumor treated with chemotherapy delivered by OAC. Moreover, the systemic exposure of the animals was 1.7% and 75% of that attained in the retina after OAC and IV infusion, respectively. Therefore, we propose the selectivity of the OAC delivery to reach the ocular structures while minimizing the probability of systemic adverse events. These findings may also imply a better ocular tumor control after OAC thereby facilitating and promoting a safer technique of enucleation.

In our animal model, it is notable that OAC attained more than 200 times higher levels of topotecan in the vitreous compared to the systemic infusion. Additionally, the vitreous-to-plasma exposure after OAC was 13 whereas it was almost 0.1 after IV infusion. Despite higher concentrations of topotecan in the vitreous along with a more selective pattern with respect to plasma after OAC, we need to reinforce two aspects of the present study. First, that we aimed at showing the advantage of OAC in delivering topotecan to the optic nerve for potential implications in patients with optic nerve involvement who are invariably enucleated [9,12]. Moreover, the disposition of a drug is not only determined by the route of administration but also by its physicochemical properties. Therefore, our results may vary to other chemotherapeutic agents used for retinoblastoma including melphalan. Interestingly, in a previous study we showed that after 1 mg of topotecan delivered through OAC in this same model, the median maximum vitreous concentration was 132ng/ml [16]. Nonetheless, in the present study the median vitreous concentration 30 minutes after the end of the infusion was almost 10 times the previously reported concentration while only exposing the eyes to 4 times the previous dose. Therefore, the vitreous exposure did not increase proportionally to the dose. This finding may be explained by a saturation of ATP-binding cassette transporters in the retinal vessels and this finding should be taken into account when deciding the dose for administration to patients [32–35]. Topotecan is a well-known substrate of at least two drug transporters, the products of ABCG2 and ABCB1 [36–38]. These proteins have been identified in the endothelial cells of retinal vessels playing a strategic role in the blood-ocular barrier by extruding substrates back to the circulation and thereby limiting the disposition of drugs to the retina and vitreous. The impact of drug dose modification in the saturation of these transporters and in the final exposure of the retina and vitreous after OAC or IV infusion should be further studied.

Adverse events associated with OAC administration or infused chemotherapy include avascular retinopathy, chorioretinal abnormalities, reduction in electroretinography responses, periocular edema and redness, loss of eyelashes, bronchospasm, allergic reactions to iodinated contrast and one case of III cranial nerve palsy [39,40,41]. In addition, grade 3 or 4 neutropenia has been previously associated with dosages of melphalan higher than 0.4 mg/kg [42,43]. In the context of such a complex technique of chemotherapy delivery, several factors influence the incidence of side effects including the expertise in developing the technique and therefore, central nervous system adverse events should be evaluated on an individual basis.

Our data have some limitations related to the model used. First, the pig is a suitable animal model for performing OAC and pharmacokinetic studies due to its size that allows for feasible catheterization of the ophthalmic artery [16,21]. The porcine vascular anatomy has been well characterized for its intended use of this model for conducting research and in interventional radiology training [23]. Nonetheless, there are differences in the blood supply to the optic nerve and ocular structures between pigs and humans and this should be regarded as a limitation of our model. Among the anatomical differences between humans and pigs, the ophthalmic artery in the pig is a continuity of the external carotid while in humans it arises from the internal carotid [16,22,23]. Moreover, in the optic nerve there is no central retinal artery but six peripheral arteries that irrigate the nerve with pial vessels [31]. Therefore, anatomical dissimilarities between species may lead to differences in drug disposition in the ocular structures including the optic nerve after OAC. In addition, the pig is a non-tumor-bearing animal model and in the clinical situation, chemotherapy concentrations in the ocular tissues may differ from the obtained in the present study due to a disruption of the blood-retinal barrier caused by the tumor as previously proposed for carboplatin in patients with retinoblastoma compared to non-tumor bearing animal model [44]. Nonetheless, the influence of tumor in the optic nerve on the disposition of chemotherapy has never been characterized before. Finally, blood supply from one eye in pigs is usually shared by both eyes.

In conclusion, in a swine model OAC with topotecan lead to higher optic nerve, retina, and vitreous levels than after intravenous delivery of the same dose. In addition, OAC was selective to the optic nerve with a plasma exposure similar to that after IV infusion and therefore, a low probability of developing systemic adverse events or manageable adverse events should be expected if administered to patients with disseminated retinoblastoma. Based on these results, our group is considering the use of OAC in the armamentarium of treatment of patients with optic nerve invasion.
